# Physiologically Motivated Sequential Population Modeling of Albumin Trends and Vedolizumab Pharmacokinetics for Pregnancy Dosing Regimen Optimization

**DOI:** 10.1002/cpt.70145

**Published:** 2025-12-14

**Authors:** Zrinka Duvnjak, Robin Michelet, Casper Steenholdt, Ella S.K. Widigson, Cæcilie Skejø, João A. Abrantes, Wilhelm Huisinga, Mette Julsgaard, Charlotte Kloft

**Affiliations:** ^1^ Department of Clinical Pharmacy and Biochemistry, Institute of Pharmacy Freie Universität Berlin Berlin Germany; ^2^ Graduate Research Training Program PharMetrX Berlin/Potsdam Germany; ^3^ Department of Medical Gastroenterology Odense University Hospital Odense Denmark; ^4^ Research Unit of Medical Gastroenterology, Department of Clinical Research University of Southern Denmark Odense Denmark; ^5^ Department of Hepatology and Gastroenterology Aarhus University Hospital Aarhus Denmark; ^6^ Department of Clinical Medicine Health, Aarhus University Aarhus Denmark; ^7^ Roche Pharma Research and Early Development, Pharmaceutical Sciences Roche Innovation Center Basel Basel Switzerland; ^8^ Institute of Mathematics, Universität Potsdam Potsdam Germany

## Abstract

The pharmacokinetics (PK) of monoclonal antibodies (mAbs) during pregnancy remains poorly characterized, despite active inflammatory bowel diseases (IBD) being the greatest risk factor for adverse pregnancy outcomes. To quantify pregnancy‐induced changes, vedolizumab concentrations from 39 pregnant patients on various dosing regimens were analyzed using a sequential albumin‐trend/PK modeling approach, extending a published vedolizumab non‐pregnancy model. Albumin trends were first characterized using a polynomial mixed‐effect model. Then, individual changes in albumin from their pre‐pregnancy concentrations, implemented as time‐varying patient‐influential factor (covariate) in the PK model, served as potential biomarker of pregnancy‐induced plasma volume expansion. The modeling framework allowed model‐informed imputation of missing covariate data, extraction of hemodilution effect, and estimation of pre‐pregnancy PK parameters. Due to albumin change, the central volume of distribution increased 52.4%, consistent with known gestational plasma volume expansion, while clearance increased to 38.6%. An additional third‐trimester effect of gestational age, potentially reflecting transplacental transfer, increased clearance by an additional 33.3 percentage points. These changes led to a 49.5% decline in vedolizumab trough concentrations (*C*_min_) by late pregnancy. To maintain efficacious pre‐pregnancy exposure (dependent on the individual dosing interval), dosing intervals were gradually shortened for approximately one‐third (e.g., to up to 5.6 weeks for pre‐pregnancy 8‐week regimens). Optimized dosing times were summarized in an easy‐to‐use nomogram‐like plot. This work provides the first population PK model of vedolizumab in pregnancy. By integrating physiologically motivated pregnancy effects, it advanced quantitative understanding of mAbs PK in pregnancy with potential application to other biologics and provides optimized dosing strategies to mitigate risks of adverse pregnancy outcomes.

## Graphical abstract

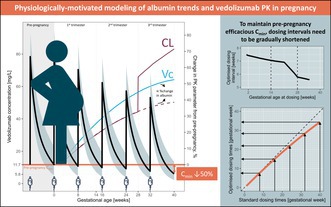

**Study Highlights**

**WHAT IS THE CURRENT KNOWLEDGE ON THE TOPIC?**

The impact of pregnancy on monoclonal antibody pharmacokinetics is sparsely studied, despite active inflammatory bowel disease being the greatest risk factor for adverse pregnancy outcomes.

**WHAT QUESTION DID THIS STUDY ADDRESS?**

What is the magnitude of the impact of individual plasma volume expansion and residual pregnancy effect on vedolizumab pharmacokinetics? What is the optimized dosing regimen that maintains efficacious pre‐pregnancy exposure?

**WHAT DOES THIS STUDY ADD TO OUR KNOWLEDGE?**

By applying covariate‐trend/pharmacokinetic modeling, the study overcame data challenges and identified individual albumin changes as a potential biomarker of gestational plasma volume expansion. Central volume of distribution was found to increase 52.4% and clearance 71.9% throughout pregnancy. Vedolizumab trough concentrations dropped by up to 49.5%, supporting the need for gradually shortened dosing intervals, for up to approximately one‐third.

**HOW MIGHT THIS CHANGE CLINICAL PHARMACOLOGY OR TRANSLATIONAL SCIENCE?**

The developed framework enhances quantitative understanding of monoclonal antibody pharmacokinetics in pregnancy with potential application to other biologics, and provides optimized dosing strategies in a nomogram‐like plot to mitigate risks of adverse pregnancy outcomes.

Understanding drug pharmacokinetics (PK) during pregnancy is crucial to ensure effective treatment and maintain disease remission, thereby reducing the risk of adverse pregnancy outcomes. However, there is a limited understanding of how pregnancy impacts the PK of small‐molecule drugs, and even less is known about monoclonal antibodies (mAb).^1^, ^2^, ^3^ While recent developments in physiologically based pharmacokinetic (PBPK) modeling of mAbs in pregnancy represent a substantial step forward, PBPK models still lack sufficient predictive accuracy for guiding dosing recommendations.^4^, ^5^ Additionally, the few published population, nonlinear mixed‐effects (NLME) PK models incorporated pregnancy solely as a categorical patient‐influential factor (covariate) of pregnancy trimesters.^6^, ^7^ This scarcity of quantitative understanding highlights the need for deeper exploration of how pregnancy influences mAbs PK.

MAbs are mostly confined to the extracellular space, with plasma being an important component, and primarily eliminated through nonspecific lysosomal degradation by endothelial cells lining the blood vessels.^8^ Depending on the dose, interactions with their targets can also play a role in mAbs’ overall disposition. Immunoglobulin G (IgG)‐based mAbs show slow elimination, resulting from the neonatal fragment crystallizable receptor (FcRn) salvage pathway.^8^ Albumin, a common covariate in NLME PK models of mAbs, is thought to reflect the efficiency of this pathway, though albumin concentrations are also influenced by inflammation status.^9^, ^10^ Other often identified covariates include body‐size descriptors, i.e., body weight.^11^

Pregnancy influences both albumin and body weight; however, their changes in pregnancy have different implications on the PK of mAbs than equivalent‐size changes in non‐pregnant populations. In non‐pregnancy, weight gain is generally linked to fat tissue, whereas in pregnancy, it is primarily attributed to fetus‐associated organs, and an increase in maternal extracellular fluid, including plasma volume.^12^, ^13^ Given that mAbs are mostly confined to extracellular space, an increase in plasma volume may have a significant impact on mAbs PK in pregnancy.^1^ Moreover, there is high interindividual variability (IIV) in the magnitude of plasma expansion and these differences may be relevant when interpreting measured biomarker concentrations.^14^ In this regard, the pregnancy‐related decrease in albumin concentrations has been hypothesized to be predominantly linked to plasma volume expansion.^1^, ^12^, ^15^ This link is further demonstrated by albumin’s common use in different methods for plasma volume determination in non‐pregnant populations.^16^ Thus albumin, one of the commonly available biomarkers in clinical practice, could offer insights into changes in the central volume of distribution (Vc) and clearance (CL) of mAbs on an individual level.

Due to the mAbs IgG nature, PK of mAbs during pregnancy is also influenced by transplacental transfer, particularly in the third trimester.^17^, ^18^ In addition, pregnancy has been shown to induce numerous other physiological changes. However, their relevance for mAb PK remains unknown.^12^, ^18^, ^19^

Developing an NLME PK model that captures pregnancy‐specific changes is further challenged by data availability. As a highly vulnerable population, in addition to children, pregnant patients are typically excluded from clinical trials with intensive PK sampling, and real‐world data are commonly sparse.^20^, ^21^ Furthermore, pre‐pregnancy PK data are rarely available, making it difficult to establish baseline (pre‐pregnancy) parameters and, therefore, quantify their change. Additionally, proposing dosing adjustments for mAbs in pregnancy is complicated by mAbs’ long dosing intervals and unpredictable pregnancy start, relative to the last administered dose. This results in dosing scheduled for different gestational ages (GAs) for different patients, each requiring a different optimized regimen.

Vedolizumab, a humanized mAb against α4β7 integrin, prevents gut‐homing T cell migration, thereby reducing inflammation, and is approved for the treatment of inflammatory bowel diseases (IBD), namely, Crohn’s disease (CD) and ulcerative colitis (UC).^22^ Vedolizumab has been shown to cross the placenta; however, accumulated evidence supports its safety for both mother and fetus in pregnancy.^23^, ^24^, ^25^ Consequently, a global consensus now also recommends continuing vedolizumab treatment throughout pregnancy.^26^ The approved vedolizumab maintenance dosing regimen is 300 mg intravenous infusion every 8 weeks (Q8W). However, in clinical practice, dosing regimen intensification (Q6W or Q4W) is applied for some patients, to reach and maintain remission when standard Q8W is not efficacious.^27^ There is a potential risk that during pregnancy, patients fall below their efficacious thresholds, since vedolizumab *C*_min_ (trough) concentrations have been reported to decline during gestation.^25^ Concerningly, active IBD was shown to be the highest risk factor for adverse pregnancy outcomes.^28^, ^29^

This study aims to: (i) quantify and understand the impact of pregnancy on vedolizumab PK by NLME modeling with incorporating physiologically motivated pregnancy‐induced effects; (ii) model‐inform an optimized dosing regimen to maintain vedolizumab individual efficacious pre‐pregnancy exposure, ultimately decreasing the risk of adverse pregnancy outcomes.^2^, ^21^, ^30^

## METHODS

### Clinical study data

Data from 39 pregnant patients with IBD (56.4% CD and 43.6% UC), receiving vedolizumab in a multicenter, prospective, observational study, were used. Baseline (pre‐pregnancy) data included disease characteristics, biomarkers of disease activity (e.g., fecal calprotectin (fcal), C‐reactive protein (CRP), and albumin concentrations), patient demographics, and therapy history. Most participants (89.7%) were on maintenance vedolizumab therapy at enrollment, while four patients (10.3%) initiated therapy during pregnancy. All patients received 300 mg vedolizumab as a 30 min intravenous infusion. The majority (69.2%) followed the standard dosing interval of Q8W, while 12.8% followed intensified Q6W and 17.9% Q4W regimens. Of 27 patients who initiated vedolizumab > 6 months before conception, 78% were in remission. Vedolizumab plasma concentrations were measured at *C*_min_ (i.e., trough concentrations, just before the next dosing), in the third trimester when the dosing was skipped and during delivery. Other longitudinal data collected included albumin, fcal, and CRP concentrations, body weight, anti‐drug antibodies (ADA), disease activity, and concomitant medications. Albumin measurements were missing for 10.9% of vedolizumab observations. In total, 167 albumin concentrations were available, including both baseline (pre‐pregnancy) and pregnancy measurements. ADA testing was performed only on samples with vedolizumab ≤ 3 mg/L, with all 12 tested samples returning negative results. In total, 138 *C*_min_ samples were collected (median [range]: 4 [2–5] per patient), spanning in GA 7–42 weeks. Two PK samples below the lower limit of quantification (LLOQ; 0.5 mg/L) were set to half the LLOQ.

#### Ethics statement

The study was approved by the Danish Data Protection Agency (1‐16‐02‐645‐16) and the Regional Ethical Review Board (1‐10‐72‐269‐16). Written informed consent was obtained from all participants.

## Sequential albumin‐trend/PK modeling

The covariate‐trend/PK model was developed in a stepwise manner, analogous to established sequential pharmacokinetic/pharmacodynamic (PK/PD) modeling, “Individual PK parameters” approach.^31^ In Step 1, the covariate‐trend model, namely, the model of albumin trends in pregnancy, was developed. In step 2, individual parameter estimates from the developed albumin‐trend model were used to predict individual percentage change in albumin from the estimated baseline throughout pregnancy and thereafter used as a time‐varying covariate on CL and Vc in the PK model. In this work, change in albumin was selected as a variable of interest since it potentially reflects one of the key processes in pregnancy relevant for mAbs PK, namely plasma volume expansion, in contrast to the increase in body weight, that lumps numerous pregnancy‐related changes, all with potentially different implications on the PK of mAbs and associated with high variability (e.g., newborn body weight, increase in fat tissue, increase in breasts, increase in plasma volume).

## Step 1: Albumin‐trend model development

Pregnancy and baseline albumin concentrations were modeled together as a function of fertilization age (FA), representing the time from conception (true pregnancy start), as it is commonly done when modeling biomarker changes throughout pregnancy.^12^, ^19^ For characterizing albumin trends in pregnancy, polynomial models of different degrees (0–3) were considered due to their flexibility and simplicity. IIV in parameters and their relations were examined and kept in the model if estimated with relative standard error (RSE) < 50%. As assumed distributions of individual parameters, normal and log‐normal distributions were assessed. Proportional, additive, and combined additive+proportional residual unexplained variability (RUV) models were tested. Baseline variables, e.g., age, diagnosis, disease activity biomarkers, and extent of change in body weight, were both graphically and formally explored as covariates.

## Step 2: Physiologically motivated PK model development

Given the PK data characteristics (sparse sampling, one dose level), the model of vedolizumab in pregnancy was built as an extension of a previously developed model from the vedolizumab clinical drug development program, referred to as the reference model: two‐compartment disposition model, with parallel linear and nonlinear elimination from the central compartment.^32^ The structural components were re‐estimated if possible. The study population and reference model population were similar, except that the study population had a lower level of inflammation and was on prior anti‐TNF‐α therapy (**Table** **1**). Since (i) all the reported parameter estimates of covariate effects in the reference model assumed the “Typical reference individual” (centering of covariate effects) being in some covariates different from the “Representative pregnancy‐study individual” (**Table** **S1**), and since, (ii) in general, all reported parameter estimates in the model will be conditioned on the presence of other parameters, it was decided to keep all the covariate effects from the reference model (e.g., prior anti‐TNF‐α therapy status, even though all the patients in our populations received prior anti‐TNF‐α therapy). Moreover, since pregnancy may have an impact on these covariate effects, all the covariates from the reference model were fixed as time‐invariant baseline (pre‐pregnancy) covariates. Derived percentage change in albumin from step 1 was tested as a time‐varying covariate on Vc and CL. The residual impact of GA as a time‐varying covariate on CL and Vc was tested as an exponential model with different GA cut‐off values for the start of the effect. GA represents the time from the last menstrual period (easy to determine), and it is commonly used in clinical practice (FA = GA−2). To aid communication of results, GA was used throughout PK modeling and simulation. Assessments of IIV were performed on all structural parameters, assuming a log‐normal distribution of individual parameters. Proportional, additive and combined additive+proportional RUV models were assessed.

**Table 1:** Comparison of the pregnancy‐study population in their baseline (pre‐pregnancy) covariate values and the reference PK model population [32]

Demographics	Pregnancy‐study population (*N* = 39)	Reference population (*N* = 2,554)
	Median [range]	Median [range]
Continuous covariates^a^		
Albumin [g/L]	41.0 [28.0, 50.0]	37 [11–53]
Body weight [kg]	69.0 [48.0, 113]	68 [28–170]
Fecal calprotectin [mg/kg]	187 [21.0, 2,870]	720 [2,375–20,000]
CDAI score	~100^b^	320 [93–580]
Partial Mayo score	~1^b^	6 [1–9]
Age [years]	30.0 [22.0, 42.0]	36 [18–78]
Categorical covariates^a^		
Presence of antidrug antibodies	Yes: 0%	Yes: 5%
	No: 100%	No: 95%
Diagnosis	CD: 56.4%	CD: 60%
	UC: 43.6%	UC: 37%
		Healthy: 3%
Prior anti‐TNF‐α therapy use	Yes: 100%	Yes: 52%
	No: 0%	No: 43%
		Missing: 5%

Abbreviations: CDAI, Crohn’s Disease Activity Index; TNF, tumor necrosis factor.

^a^ Covariate effects are ordered by the effect size within the data type category.

^b^ Not assessed, approximation made by gastroenterologist.

## Model evaluation

All models were evaluated using goodness of fit (GoF) plots, difference in objective function value (dOFV), parameter precision, and plausibility of parameter estimates.^33^ The key models were also assessed using prediction‐corrected visual predictive check (pcVPC) (*n* = 2,000), and parameter precision using sampling importance resampling (SIR).^34^, ^35^

## Simulation‐based analysis of pregnancy effects

The impact of pregnancy on PK parameters and vedolizumab concentrations was evaluated through deterministic and stochastic simulations (*n* = 2,000) for a representative pregnancy‐study individual and population (**Table** **S1**) using both univariate and multivariate approaches. Scenarios with varying timings of pregnancy onset within the dosing interval were simulated. For the analysis of changes in vedolizumab *C*_min_ throughout pregnancy, data from all scenarios were combined.

## Dosing regimen optimization

The dosing regimen (i.e., dosing interval) was optimized if the simulated decrease in the *C*_min_ from pre‐pregnancy exceeded 20%.^36^ The optimization was performed for the representative pregnancy‐study individual (**Table** **S1**) and the three commonly used maintenance dosing intervals in clinical practice: Q8W, Q6W, and Q4W (one of which was efficacious for the individual patient in pre‐pregnancy). The optimized dosing times (patient’s GA) were derived using iterative simulations from the developed pregnancy vedolizumab PK model, administering the next dose when vedolizumab concentrations dropped below its pre‐pregnancy *C*_min_ values (given by the model) (**Figure** **S1a**). This was done separately for eight scenarios, differing in the time of pregnancy onset within dosing intervals (**Figure** **S1b**, left).

To summarize optimized dosing times derived in the previous step, optimized dosing times were modeled together as a polynomial function of the corresponding standard, scheduled dosing times (assuming pre‐pregnancy dosing intervals were maintained throughout pregnancy; **Figure** **S1b**, right).

## Software

PsN v5.0.0 (https://uupharmacometrics.github.io/PsN/) was used to access NONMEM v7.5.1 (ICON Development Solutions, Hanover, MD) through Pirana v24.9.2 (Certara, Princeton, NJ). Model parameters were estimated using the first‐order conditional estimation with interaction method. Simulations of vedolizumab PK were performed using rxode2 v.3.0.1 (https://nlmixr2.github.io/rxode2/). Data management, data visualization, and processing of modeling results were performed using R v4.4.2 with RStudio v2024.09.0+375 (https://posit.co/download/rstudio‐desktop/).

## RESULTS

### Albumin‐trend model

**Figure 1:**
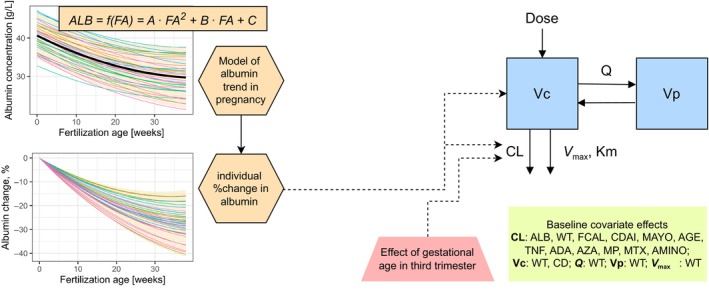
Scheme of integrated albumin‐trend and physiologically motivated pharmacokinetic (PK) model of vedolizumab in pregnancy. The albumin trend was modeled as a second‐order (quadratic) polynomial function of fertilization age (FA). Upper left: estimated typical prediction (thick blue line) and individual albumin predictions for study patients (colored thin lines); Bottom left: derived individual percentage change in albumin for study patients. The background light orange bands span over 90% simulated profiles (*n* = 2,000). The developed vedolizumab PK model in pregnancy (right) was a two‐compartment disposition model with parallel linear and nonlinear elimination from the central compartment, time‐varying effect of individual predicted percentage change in albumin on CL and Vc, and time‐varying effect of gestational age in the third trimester on CL. Baseline covariate effects were fixed to the estimates from the reference model [32]. ADA, anti‐drug antibody; ALB, albumin concentration; AMINO, aminosalicylate; AZA, azathioprine; CD, Crohn’s disease; CDAI, Crohn’s disease activity index; CL, clearance; FCAL, fecal calprotectin; Km, concentration of vedolizumab at half‐maximum elimination rate; MAYO, partial Mayo score; MP, 6‐mercaptopurine; MTX, methotrexate; Q, intercompartmental clearance; TNF, tumor necrosis factor‐α; Vc, central volume of distribution; *V*_max_, maximum elimination rate; Vp, peripheral volume of distribution; WT, body weight.

For characterization of individual albumin trajectories, the second‐order (quadratic) polynomial, $\mathrm{ALB}=f\left(\mathrm{FA}\right)$ $=A\cdot {\mathrm{FA}}^{2}+B\cdot \mathrm{FA}+C$, was found appropriate. The baseline albumin parameter (parameter C) was estimated to be 40.7 g/L (**Table** **2**), closely aligning with the observed population median of 41.0 g/L. Estimates of linear and quadratic coefficients (parameters B and A) described a typical decrease in albumin concentrations from 40.7 g/L at the beginning of pregnancy (FA = 0 or GA = 2) to 29.7 g/L at the end of pregnancy (FA = 38 or GA = 40), corresponding to a reduction of 26.9% (90% simulation interval: 13.5%–41.4%) (**Figure** **1**, left). The extent of albumin reduction (estimates of individual linear coefficient parameters, parameter B) was not found to correlate with changes in body weight, or baseline body weight, albumin, CRP, fcal, age, diagnosis, or disease duration (**Figure** **S2**).

**Table 2:** Parameters of the developed albumin‐trend model and physiologically motivated pharmacokinetic (PK) model of vedolizumab in pregnancy

Parameter		Estimate (RSE^a^, %)	95% confidence interval^a^
Model of albumin‐trends in pregnancy, $\mathrm{ALB}=f\left(\mathrm{FA}\right)=A^{2}\cdot \mathrm{FA}+B\cdot \mathrm{FA}+C$			
Base model parameters			
A	Quadratic coefficient	0.0059 (23)	[0.00328 to 0.00837]
B	Linear coefficient	−0.511 (9)	[−0.598 to −0.414]
C	Y‐intercept (baseline albumin)	40.7 (2)	[39.3 to 41.9]
Interindividual variability (IIV) parameters			
IIV_B^b^, ^c^	Interindividual variability in B, SD	0.0860 (44)	[0.0556 to 0.123]
IIV_C^b^, ^c^	Interindividual variability C, SD	3.69 (29)	[2.85 to 4.84]
Residual unexplained variability (RUV) parameters			
RUV_ADD^c^	Additive RUV, SD [g/L]	2.06 (15)	[1.77 to 2.40]
Physiologically motivated PK model of vedolizumab in pregnancy			
Base model parameters^d^			
CL	Reference UC clearance, L/day	0.159 (fixed)	—
	Reference CD clearance, L/day	0.155 (fixed)	—
Vc	Central volume of distribution, L	4.33 (8.0)	[3.63 to 5.00]
Vp	Peripheral volume of distribution, L	1.65 (fixed)	—
Q	Intercompartmental clearance, L/day	0.12 (fixed)	—
*V*_max_	Maximum elimination rate, mg/day	0.265 (fixed)	—
Km	Concentration at half‐maximum elimination rate, mg/L	0.964 (fixed)	—
Pregnancy covariate effects			
DALBCL^e^	Effect of estimated albumin change on CL	−1.44 (22)	[−2.17 to −0.861]
DALBVC^e^	Effect of estimated albumin change on VC	−1.96 (35)	[−3.52 to −0.770]
T3GACL^f^	Effect of gestational age in the 3rd trimester on CL	0.151 (27)	[0.0728 to 0.236]
Baseline covariate effects^g^			
WTCL	Body weight on CL	0.362 (fixed)	—
ALBCL	Albumin on CL	−1.18 (fixed)	—
FCALCL	Fecal calprotectin on CL	0.0310 (fixed)	—
CDAICL	CDAI score on CL	−0.0515 (fixed)	—
MAYCL	Partial Mayo score on CL	0.0408 (fixed)	—
AGECL	Age on CL	−0.0346 (fixed)	—
WTVC	Body weight on VC	0.467 (fixed)	—
WTVP	Body weight on VP	1 (fixed)	—
WTVMAX	Body weight on *V*_max_	0.75 (fixed)	—
WTQ	Body weight on *Q*	0.75 (fixed)	—
TNFCL	Prior TNF‐α antagonist therapy status on CL	1.04 (fixed)	—
ADACL	Antidrug antibody status on CL	1.12 (fixed)	—
AZACL	Azathioprine use on CL	0.998 (fixed)	—
MPCL	6‐mercaptopurine use on CL	1.04 (fixed)	—
MTXCL	Methotrexate use on CL	0.983 (fixed)	—
AMINOCL	Aminosalicylate use on CL	1.02 (fixed)	—
CDVC	CD diagnosis	1.01 (fixed)	—
Interindividual variability parameters			
IIV_CL^c^, ^h^	Interindividual variability in CL, %CV	19.0 (23)	[15.2 to 24.0]
Residual unexplained variability (RUV) parameters			
RUV_PRO^c^	Proportional RUV, %CV	17.1 (18)	[14.6 to 20.8]
RUV_ADD^c^	Additive RUV, SD [mg/L]	1.11 (43)	[0.766 to 1.61]

Abbreviations: ADA, anti‐drug antibodies; ALB, albumin concentration; (B)WT, (baseline) body weight; CD, Crohn’s disease; CDAI, Crohn’s Disease Activity Index; DALBIPRED, individual predicted %change in albumin concentration from its pre‐pregnancy value; FCAL, fecal calprotectin; GA, gestational age; MAY, Mayo score; RSE, relative standard error; SD, standard deviation; TNF, tumor necrosis factor‐α; TV, typical value; UC, ulcerative colitis; *θ*, parameter estimate.

^a^ Calculated using the sampling‐importance resampling (SIR) approach.

^b^ Individual parameters assumed to be normally distributed.

^c^ Shrinkage estimates: IIV_B: 32%, IIV_C: 7.0%, RUV_ADD (albumin): 17%, IIV_CL: 19%, RUV_PRO: 13%, RUV_ADD (vedolizumab): 13%.

^d^ Typical individual: **Table** **S1**.

^e^ Albumin effects were modeled using a linear relationship, e.g., DALBCLEFF = 1 + DALBIPRED·*θ*_DALBCL_; DALBIPRED = 100·(ALB−*C*)/*C*.

^f^ Effect of 3rd trimester gestational age was modeled using an exponential relationship, e.g., T3GACLEFF = *e* ^(GA/28)·^ *^θ^* ^_T3GACL^.

^g^ Baseline covariate effects were modeled using power relationships, e.g., WTCLEFF = (BWT/reference_BWT_) *^θ^* ^_WTCL^;for continuous covariates with example of BWT and ADACLEFF = *θ*_ADA_^STATUS_ADA^ for categorical covariates with example of ADA status, typical parameters were calculated by multiplying the parameter estimate and covariate effect terms, e.g. TVCL = *θ*_CL_·ADACLEFF·WTCLEFF·T3GACLEFF·DALBCLEFF … The reader is referred to the Supplementary materials for the full model code.

^h^ Individual parameters assumed to be log‐normally distributed.

**Figure 2:**
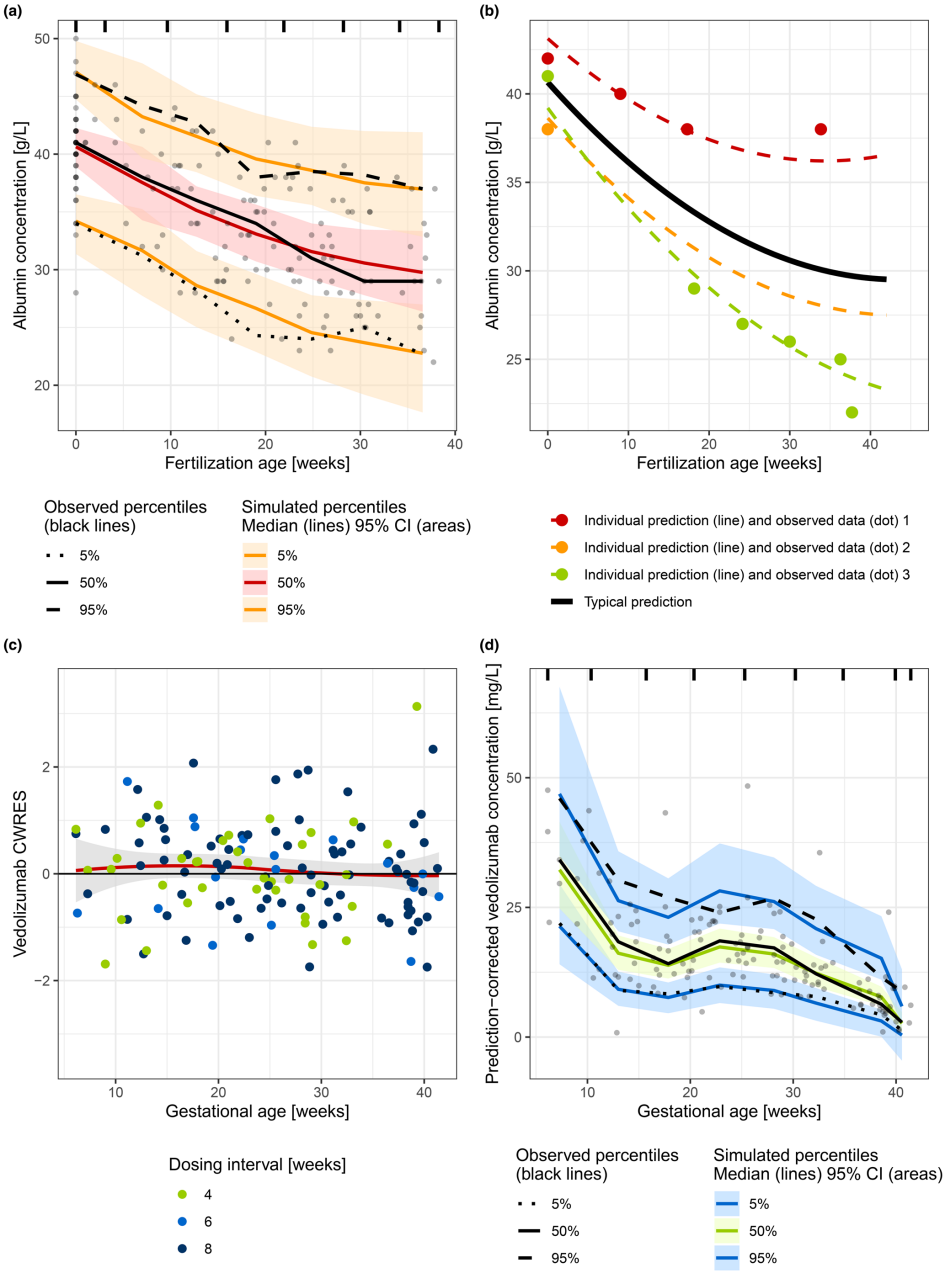
Model diagnostics for the albumin‐trend model (**a, b**) and physiologically motivated pharmacokinetic (PK) model of vedolizumab in pregnancy (c and d). (**a**) Visual predictive check (VPC) of the developed albumin‐trend model (*n* = 2,000). Gray points represent observed albumin concentrations. Ticks on the top represent bin borders. (**b**) Capability of the polynomial model to capture different trends in albumin concentration‐fertilization age profiles. Typical albumin prediction (thick black line) and three extreme examples of measured albumin data (dots) and individual predictions (dashed colored lines) of the polynomial albumin‐trend model are shown. Individual one (red): modest change, individual two (orange): only pre‐pregnancy albumin measurement available, individual three (green): prominent change in albumin concentrations. (**c**) Goodness of fit plot: conditional weighted residuals (CWRES) vs. gestational age (GA) stratified by maintenance dosing interval. One data point at GA = 12.9 and CWRES = ‐5.66 is not shown in the plot (note: it was included in the estimation of model parameters). The red line represents a locally estimated scatterplot smoothing (LOESS) fit with a gray shaded 95% confidence interval. (**d**) Prediction‐corrected visual predictive check (pcVPC) of the developed physiologically motivated PK model of vedolizumab in pregnancy (*n* = 2,000). Gray points represent observed (prediction‐corrected) vedolizumab concentrations. Ticks on the top represent bin borders.

The polynomial model adequately captured both the population and individual albumin trends, with no misspecifications observed (**Figure** **2** **a,b** and **Figure** **S3**). All parameters were estimated with good precision (RSE ≤ 23% for fixed effects and ≤ 44% for random effects; **Table** **2**).

### Physiologically motivated PK model of vedolizumab in pregnancy

The developed PK model was a two‐compartment disposition model with parallel linear and nonlinear elimination from the central compartment, with a time‐varying covariate effect of predicted individual percentage change in albumin on CL (DALBCL: “delta albumin on CL”) and Vc (DALBVc: “delta albumin on Vc”) (dOFV: −26), and, in addition, the time‐varying covariate effect of GA in the third trimester on CL (T3GACL, i.e., GA of 28 weeks was identified as the best starting point of effect, coinciding with the start of the third trimester) (dOFV: −14) (**Figure** **1**, right). IIV was estimated on CL (19%) and RUV was described by an additive + proportional model (**Table** **2**). The model accurately predicted the data for all dosing regimens, as evidenced by the GoF plots, which showed no indication of model misspecification (**Figure** **2c** and **Figure** **S4**). Additionally, the pcVPC and covariate pcVPC plots demonstrated a good agreement between observed data and model simulations (**Figures** **2** **d** and **Figure** **S5**). All parameters were precisely estimated (**Table** **2**).

Model parameters of the peripheral disposition compartment and the nonlinear elimination parameters were fixed to the reference model values, while CL and Vc were initially re‐estimated. The Vc was estimated to be 4.33 L. Re‐estimation of CL did not significantly reduce the OFV (dOFV: −0.5) and the CL estimate was close to the reference CL value; however, estimation of CL significantly reduced the precision of other parameter estimates, leading to CL being kept fixed.

**Figure 3:**
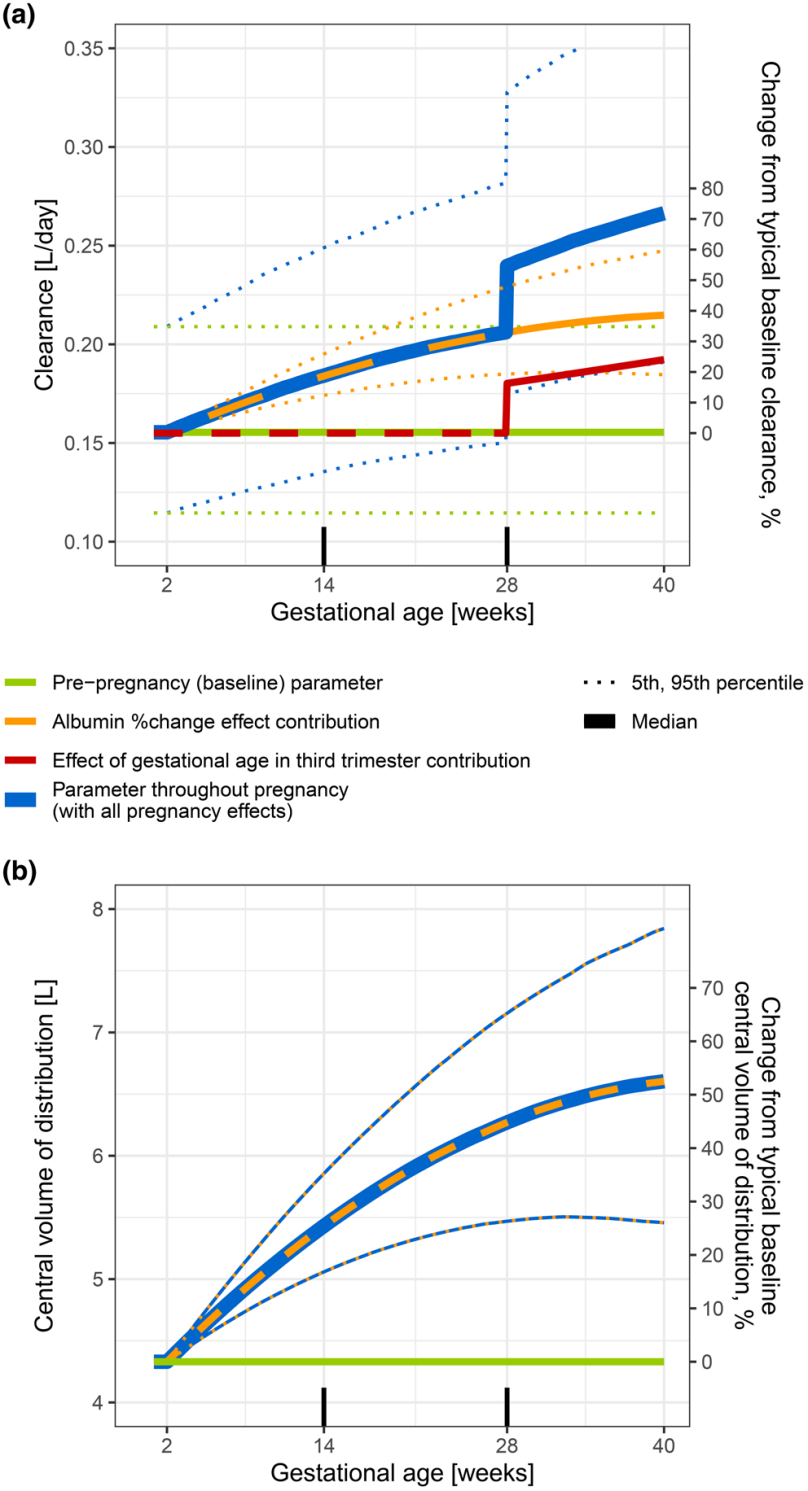
Impact of pregnancy on vedolizumab pharmacokinetic parameters throughout gestation: (i) albumin percentage change effect (orange lines), (ii) effect of gestational age in the third trimester (red line), and (iii) both effects (blue line). Pharmacokinetic values are displayed on both the absolute scale (left *y*‐axis) and relative scale (right *y*‐axis), with median values (full line), 5th and 95th percentile of simulated profiles (dotted lines, *n* = 2,000). (**a**) clearance and (**b**) central volume of distribution. Ticks above the *x*‐axis split the pregnancy into trimesters.

**Figure 4:**
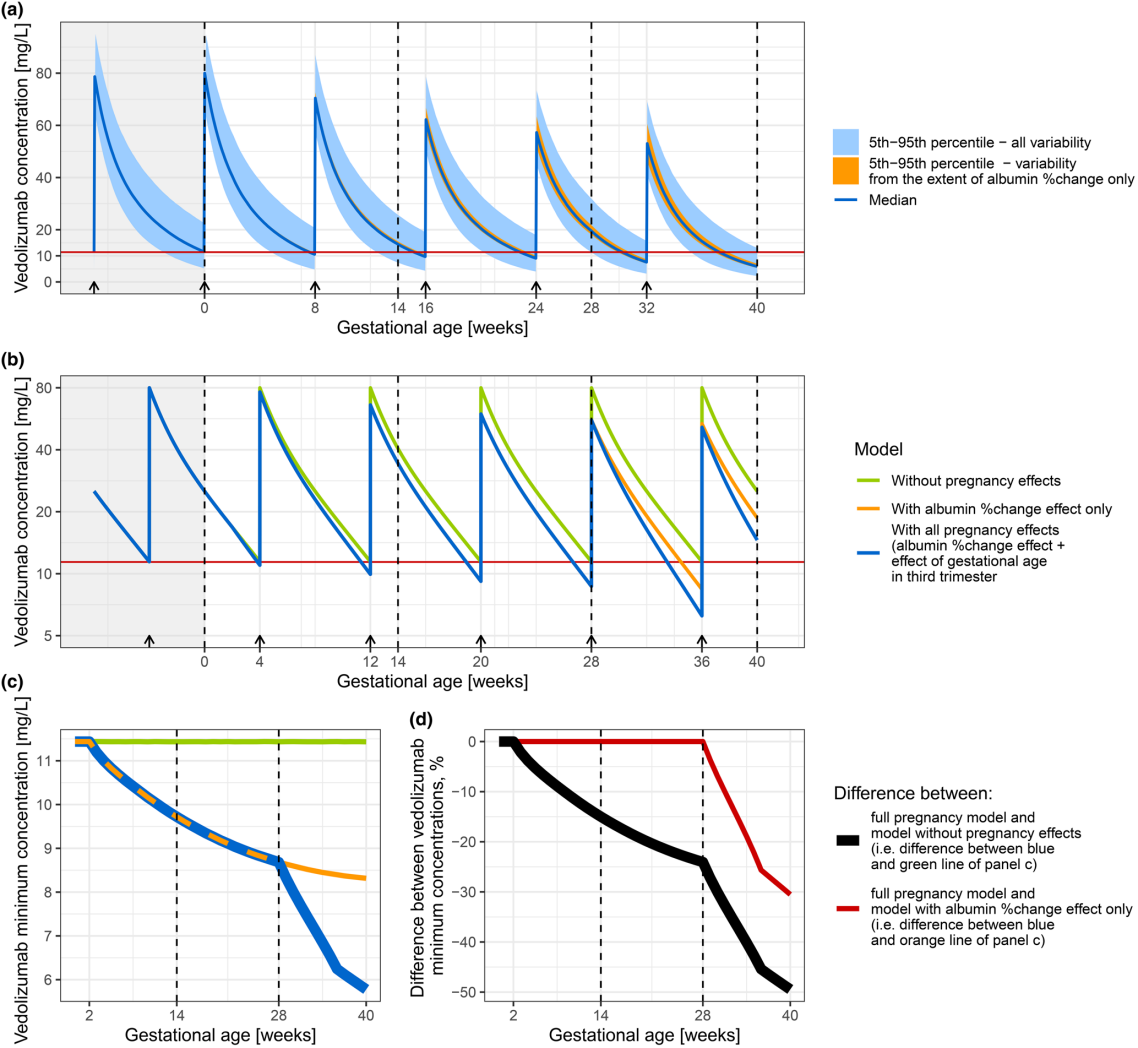
Impact of pregnancy on vedolizumab concentrations throughout pregnancy (gestational age), for a standard maintenance dosing regimen (dosing every 8 weeks). (**a**) Vedolizumab pregnancy concentration‐time profile simulated from the developed model of vedolizumab in pregnancy (*n* = 2,000), for a scenario when the first pregnancy dose was administered at a gestational age of 0 weeks. The blue line represents the population median, and ribbons around it cover 90% of the simulated data: (i) blue ribbon summarizing simulations with interindividual variability originating from clearance, baseline body weight, baseline albumin, and extent of decrease in albumin (other covariate effects were assumed to be of the representative pregnancy‐study individual – **Table** **S1**); (ii) orange ribbon summarizing simulations with interindividual variability originating only from extent of decrease in albumin. Residual unexplained variability was not simulated. The arrows above the *x*‐axis depict vedolizumab administration times. The gray area to the left represents a pre‐pregnancy state. The horizontal red line: typical vedolizumab pre‐pregnancy minimum (trough) concentration (*C*_min_) of 11.4 mg/L. Vertical dashed lines represent trimester borders. (**b**) Vedolizumab concentration‐time profile on a log‐transformed *y*‐axis, for a representative pregnancy‐study individual that received a first pregnancy dose at a gestational age of 4 weeks, simulated from (i) model without pregnancy effects (green line), (ii) model with only albumin percentagechange effect (orange line), and with all pregnancy effects (including also effect of gestational age in third trimester, i.e., developed model of vedolizumab in pregnancy – blue line). Horizontal red line: typical vedolizumab pre‐pregnancy *C*_min_ of 11.4 mg/L. (**c**) Vedolizumab *C*_min_ for a representative pregnancy‐study individual, simulated from (i) model without pregnancy effects (green line), (ii) model with albumin percentage change effect (orange line), and with all pregnancy effects (including also the effect of gestational age in the third trimester, i.e., developed model of vedolizumab in pregnancy – blue line). The lines were obtained by combining extracted *C*_min_ from scenarios that differ in the timing of the first pregnancy dose (given from gestational week 1 to 8). (**d**) total change in *C*_min_ from pre‐pregnancy throughout pregnancy (black line), and percentage contribution of the effect of gestational age in the third trimester to the changes in *C*_min_ (red line).

The impact of pregnancy on CL and Vc, including their simulated variability throughout pregnancy, revealed that Vc at the end of pregnancy typically increased to 6.60 L, which corresponded to an increase of 52.4% (90% simulation interval: 5.46–7.84 L and 26.1%–81.1%, respectively; **Figure** **3**, bottom). As a result of a decrease in albumin concentrations, by late pregnancy the typical CL increased by 38.6% (90% simulation interval: 19.1%–59.6%). At the end of pregnancy, with the additional effect of GA (an additional 33.3 percentage points increase from the CL value already impacted by the albumin change), the typical total CL increased to 0.266 L/day (71.9% increase from pre‐pregnancy). Variability in CL (**Figure** **3** **a**) was mostly attributed to the baseline clearance variability (IIV_CL), i.e., the area of the 90% simulation interval was larger for baseline CL (green dotted lines) than for albumin change contribution (orange dotted lines). This was also shown on the vedolizumab concentration level in **Figure** **4** **a**, in which the orange ribbon depicting variability originating from the extent of albumin percentage change was narrower compared to the blue ribbon depicting total variability (also including differences in baseline albumin and body weight).

As a result of changes in PK parameters, exposure to vedolizumab decreased throughout pregnancy, both in *C*_max_ and *C*_min_ (**Figure** **4** **a,b**): typical *C*_min_ decreased by 15.0% (*C*_min_: 9.70 mg/L), 24.0% (8.68 mg/L), and 49.5% (5.78 mg/L) by the end of the first, second, and third trimesters, respectively (**Figure** **4c,d**, black and blue line, respectively). The effect of GA in the third trimester contributed to a 30.5% point decrease (**Figure** **4** **d**, red line).

### Dosing regimen optimization

**Figure 5:**
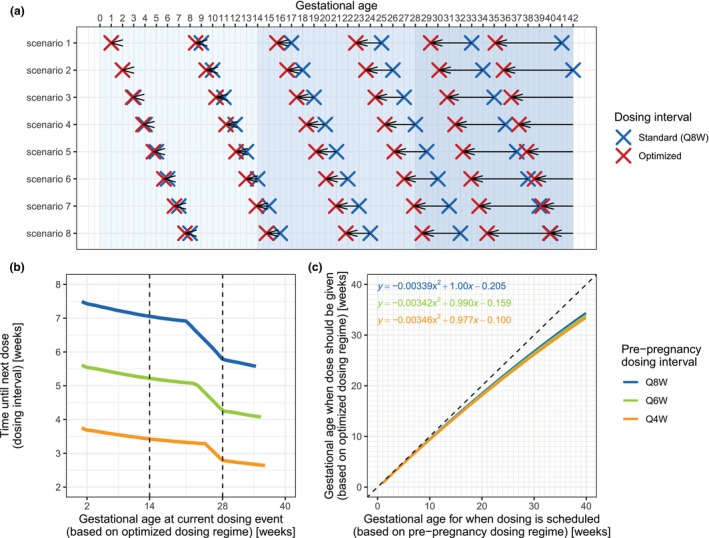
Optimized vedolizumab maintenance dosing regimens for use throughout pregnancy for a representative pregnancy‐study individual (**Table** **S1**). (**a**) Comparison of the standard maintenance (Q8W) and optimized dosing regimens, for eight scenarios that differ in pregnancy start relative to last dose, i.e., timing of first pregnancy dose (from week 1 to week 8). The blue crosses depict the dosing events based on the standard (Q8W) dosing regimen (i.e., distance between two adjacent blue crosses: 8 weeks). The red crosses depict the dosing events based on the optimized dosing regimen (distance between two adjacent red crosses: < 8 weeks). The black arrows span from standard (Q8W) to optimized dosing events. Different shades of background blue color represent different pregnancy trimesters. (**b**) Optimized dosing intervals throughout pregnancy for pre‐pregnancy Q8W, Q6W, or Q4W dosing regimens (i.e., the distance between the red crosses from panel **a**)). The vertical dashed lines represent pregnancy trimester borders. (**c**) Nomogram‐like plot summarizing optimized dosing regimen. Relationship between gestational age at dosing events based on the optimized dosing regimen (e.g., for Q8W: red crosses from panel **a**) and gestational age at dosing events based on the standard pre‐pregnancy (scheduled) dosing regimen (e.g., for Q8W: blue crosses from panel **a**) was described by a second‐order polynomial function. The functions or the plot itself can be used for the determination of the optimized dosing regimen for any given scheduled time set. Q4W, every 4 weeks; Q6W, every 6 weeks; Q8W, every 8 weeks.

Based on simulations from the developed model, maintaining individual efficacious pre‐pregnancy vedolizumab *C*_min_ during gestation required doses to be administered progressively earlier than scheduled (based on the pre‐pregnancy dosing interval) as pregnancy advanced (**Figure** **5** **a**). For the common pre‐pregnancy dosing regimens of Q8W, Q6W, and Q4W, the duration of optimal dosing intervals gradually decreased up to (at the end of pregnancy) for approximately one‐third, compared to the pre‐pregnancy dosing interval duration, i.e., to dosing intervals 5.6, 4.1, and 2.6 weeks long, respectively (**Figure** **5** **b**).

For calculating the patient’s GA at which the dose should be administered (based on the optimized dosing regimen), a nomogram‐like plot and polynomial functions were derived (**Figure** **5** **c**). For use in practice, only scheduled dosing times (if the pre‐pregnancy dosing regimen would be maintained) are needed. For example, for a patient on a pre‐pregnancy Q8W regimen, scheduled dosing at GA 2, 10, 18, 26, 34, and 42 weeks would correspond to optimized dosing times of approximately 1.8, 9.5, 16.7, 23.5, 29.9, and 35.8 weeks of gestation, respectively.

## DISCUSSION

In this work, a physiologically motivated NLME PK model of vedolizumab in pregnancy, which utilized individual albumin changes as a potential hemodilution biomarker, was successfully developed and used to derive an optimized dosing regimen of vedolizumab throughout pregnancy, summarized in a nomogram‐like plot.

The developed population covariate‐trend/PK framework—an adaptation of the sequential PK/PD modeling strategy and another integrative model—offered several advantages.^37^ In Step 1, the mixed‐effects covariate‐trend (albumin) model was developed. Therefore, the covariate data, later used in the PK model (both baseline albumin effect and time‐varying percentage albumin change), were not assumed to be error‐free, as with the standard use of covariate effects, but rather both the IIV and RUV were explicitly accounted for, analogous to the recent concept available in the full random‐effects modeling (FREM) approach.^38^ In addition, as albumin concentrations are influenced by the patient’s inflammatory status, sudden fluctuations due to changes in disease activity were accounted for by RUV, aiding in the extraction of the hemodilution effect (e.g., **Figure** **2** **b**, red individual: last observed albumin concentration was higher than the model prediction; however, the patient also experienced a decrease in CRP at the same time point). Moreover, a defined time‐varying covariate model allowed the exploration of covariates and enabled the implicit imputation of missing data using both the population‐level trends and individual‐specific observations. This was especially important for individuals for whom only baseline (pre‐pregnancy) albumin concentration was available, where the assumption of typical changes in albumin was more probable to be accurate than the assumption of no changes in albumin at all (**Figure** **2** **b**, orange individual).^39^ In Step 2, the percentage change in albumin was derived for each individual as a potential biomarker of percentage increase in plasma volume and used as a covariate on CL and Vc in the PK model. This way, the resulting size of pregnancy impact on PK was individualized, which was deemed more plausible than the assumption of an identical increase in plasma volume (also Vc and CL) for each individual would be.^40^ The developed model contained both the covariate effect of time‐invariant baseline (pre‐pregnancy) albumin (interpreted as inflammation status and efficiency of FcRn recycling) and the time‐varying covariate effect of percentage change in albumin (interpreted as pregnancy effect). The observed decrease in albumin concentrations for patients who were transitioning from active disease to remission during pregnancy (patients who started vedolizumab therapy < 6 months prior to conception), and a reported similar albumin decrease in healthy pregnancies, supported the assumption that the modeled albumin trend indeed represented the impact of pregnancy.^12^, ^19^ Finally, the implementation of time‐varying percentage albumin change also allowed for the estimation of pre‐pregnancy Vc, even though the pre‐pregnancy vedolizumab data were not available.

Pre‐pregnancy Vc was estimated to be 4.43 L–higher than the 3.19 L reported in the reference model, but in line with other published estimates, 4.62 and 4.92 L.^41^, ^42^ The resulting higher pre‐pregnancy vedolizumab *C*_min_ in our study population, compared to the reference PK model population (11.7 and 8.9 mg/L, respectively, given the representative pregnancy‐study individual), could potentially be attributed to the not fully accounted‐for difference in the inflammation status between the populations: the pregnancy population was mostly in remission with low inflammation biomarkers, while the population of the reference model mostly had higher disease activity.

The polynomial function describing albumin concentration decrease in pregnancy captured individual profiles well. As a consequence of albumin decrease by the end of pregnancy, Vc was estimated to increase 52.4%. This magnitude and trajectory of increase were in agreement with the reported plasma expansion in pregnancy.^12^, ^19^, ^43^ The relevance of an increase of Vc in pregnancy for mAbs PK was also recently recognized elsewhere, using allometric scaling and an empirical equation for the increase in plasma volume to extrapolate from non‐pregnant to pregnant population.^44^ The increase in body size descriptors is commonly followed by an increase in CL.^45^, ^46^ This was also shown in our study, where a 52.4% increase in Vc was followed by a 38.6% increase in CL, due to the albumin‐change effect. The change in clearance (CL) may result from vascular adaptations during pregnancy, including an increase in blood vessel diameter (to store a higher volume of plasma) and placental angiogenesis, which together expand the endothelial surface area responsible for mAb degradation.^8^, ^47^ Moderate IIV in the magnitude of increase in Vc and CL associated with albumin percentage changes was observed (26.1%–81.1% and 18.5%–24.7%, respectively); however, an assessment of potential covariates of this magnitude did not reveal any associations. Nonetheless, the resulting variability on vedolizumab *C*_min_ originating from the extent of albumin percentage change was not substantial (**Figure** **4** **a**, orange variability around the blue median). The analysis of the last pregnancy effect estimated in this model—the impact of GA in the third trimester—revealed that this effect contributed to an additional *C*_min_ drop from 0% (week 28) to 30.5% (week 40) (**Figure** **4** **d**, red line). Similarly, a reduction of maternal endogenous IgG concentrations has been reported to occur during the third trimester, ultimately resulting in approximately a 25% decrease in IgG concentrations at the end of pregnancy, predominantly caused by transplacental transfer.^17^ Given that vedolizumab is IgG‐based and known to cross the placenta, this additional increase in CL may reflect transplacental transfer.^23^, ^24^, ^25^

The decision to consider a certain covariate clinically relevant and to optimize the dosing regimen is often based on the rule of 20% change in exposure.^36^ Despite area under the curve (AUC) being a more comprehensive measure of vedolizumab exposure than *C*_min_ used in clinical practice, AUC calculation in our study would have heavily depended on assumptions, as most samples were collected at trough. Our results showed that the typical decrease in *C*_min_ from pre‐pregnancy was 49.6% by the end of pregnancy, which could warrant the need for dosing regimen optimization if a constant *C*_min_ is sought. Since dosing interval adjustments would be more efficient than the increase of the dose itself, and vedolizumab is only available as a fixed 300 mg dose, the regimen was optimized by adjusting the dosing interval.^20^ Given that 30.8% of patients in our study, based on gastroenterological‐symptom‐led clinical decision, required intensified dosing pre‐pregnancy (Q4W or Q6W, instead of Q8W) to achieve and maintain remission, we aimed to optimize dosing intervals during pregnancy, so patients would maintain their pre‐pregnancy efficacious exposure. Moreover, the duration of the dosing interval was tested, but was not found to be a statistically significant covariate on pre‐pregnancy CL, supporting the hypothesis that the need for pre‐pregnancy dosing regimen intensification was not due to PK, but rather PD reasons (different patients required different exposure for efficacy).

Simulation results revealed that the dosing interval should be gradually shortened to up to 5.6, 4.1, and 2.6 weeks for patients on pre‐pregnancy Q8W, Q6W, and Q4W regimens, respectively, corresponding to approximately a one‐third decrease in duration. The additional challenge in finding a universal pregnancy‐optimized dosing regimen lies in the fact that pregnancy can begin at any time within a several‐week‐long dosing interval, requiring a unique proposal for each possible pregnancy initiation time. Assuming a representative pregnancy‐study individual, this was addressed by summarizing optimized dosing times from multiple scenarios (differing in timing of pregnancy onset) in nomogram‐like plots (and/or simple equations), allowing streamlined determination of optimized regimens based on dosing schedules. The resulting equations/plots were similar for different pre‐pregnancy dosing regimens, indicating that the use of one plot or one equation may be sufficient.

Although advancing our knowledge, this study has limitations. First, processes beyond hemodilution may contribute to albumin changes during pregnancy, and evaluation of alternative biomarkers of plasma expansion may provide deeper insights.^48^, ^49^ The lack of pre‐pregnancy vedolizumab data necessitated reliance on the albumin‐trend model for estimating pre‐pregnancy structural PK model parameters, underscoring the need, even though challenging, to collect drug‐concentration data also in women planning pregnancy. The availability of mostly *C*_min_ after flat vedolizumab dose constrained the ability to fully characterize the PK of vedolizumab in pregnancy using the study data alone, and necessitated assuming that parameters other than CL and Vc were unaffected by pregnancy. However, given that vedolizumab is registered as a flat dose and the inconvenience of sampling throughout the dosing interval in clinical practice, reliance on the Rosario *et al*., “reference model,” was observed even in other non‐pregnancy vedolizumab PK analyses.^32^, ^41^, ^42^ Validation of our findings in larger cohorts with multiple samples per dosing interval is needed. Lastly, before the implementation of an optimized dosing regimen in clinical practice, the clinical relevance of the vedolizumab exposure drop should be explored, and the final clinical decision should consider multiple factors.

In future, the dosing regimen could also be fully individualized using the developed pregnancy model together with vedolizumab and albumin concentrations within a model‐informed precision dosing (MIPD) framework.^50^ The presented modeling and simulation approach may also be extended to other mAbs and expanding the model into a middle‐out framework with additional mechanistic insights could enhance drug development in pregnancy. Lastly, the model could also be extended to characterize PK changes during the postpartum “recovery period”.

In summary, the presented model is, to our knowledge, the first empirical model to characterize the impact of pregnancy using continuous covariates potentially reflecting key physiological processes, e.g., plasma volume expansion via albumin change, and transplacental transfer, therefore advancing quantitative understanding of mAb PK in pregnancy. The developed covariate trend/PK framework effectively addressed challenges such as missing time‐varying covariate data and hemodilution effect extraction while accounting for individual differences in the magnitude of pregnancy effects. Finally, this work provides actionable insights through optimization of the dosing regimen, summarized in a nomogram‐like plot. This tool enables clinicians to easily derive optimized dosing schedules to maintain pre‐pregnancy individual efficacious vedolizumab exposure, potentially reducing the risk of disease flare and associated adverse pregnancy outcomes.

## FUNDING

This investigator‐initiated study was supported by unrestricted grants from the Health Research Fund of the Central Denmark Region, Colitis‐Crohn Denmark, the A.P. Moeller Foundation of the Advancement of Medical Science, and Takeda Pharma A/S (grant no. IISR‐2017‐102375). The external funders had no involvement in any aspect of the study and writing of the report. M.J. is supported by the NOVO Nordisk Foundation (grant no. NNF23OC0081717).

## CONFLICTS OF INTEREST

Z.D., R.M., Cæ.S., E.S.K.W., J.A.A. have no conflicts of interest. Ca.S. is a speaker for Janssen and Bristol Myers Squibb, and a consultant for Takeda. W.H. received research grants from an industry partner consortium (AbbVie, AstraZeneca, Boehringer Ingelheim, F. Hoffmann‐La Roche, Merck, Novo Nordisk and Sanofi) for the graduate research training program PharMetrX, outside the submitted work. M.J. has received a research grant from Takeda Pharma A/S supporting the current study (grant no. IISR‐2017‐102375); consulting/advisory board fees from Ferring, Takeda, AbbVie, PharmaCosmos, Eli Lilly and Tillots Pharma; speaker’s fees from Tillotts Pharma, MSD, Ferring, Janssen, and Takeda. C.K. received research grants from an industry partner consortium (AbbVie Deutschland, AstraZeneca, Boehringer Ingelheim, F. Hoffmann‐La Roche, Merck, Novo Nordisk and Sanofi) for the graduate research training program PharMetrX, from H2020‐EU.3.1.3 (FAIR) and Diurnal Ltd, all outside the submitted work.

## AUTHOR CONTRIBUTIONS

All authors wrote the manuscript. Z.D., R.M., M.J., and C.K. designed the research. Z.D., R.M., Ca.S., E.S.K.W., Cæ.S., J.A.A., W.H., M.J., and C.K. performed the research. Z.D. analyzed the data.

## Supporting information


Data S1
