# Supplementary material for: Physiologically Motivated Sequential Population Modeling of Albumin Trends and Vedolizumab Pharmacokinetics for Pregnancy Dosing Regimen Optimization
Source: Clin Pharmacol Ther. 2025 Dec 14;119(2):457–69. doi: 10.1002/cpt.70145 (PMC12816427; doi:10.1002/cpt.70145)
Supplement: Supplementary file 1 — Data S1 [file CPT-119-457-s001.docx]

**Physiologically-motivated sequential population modeling of albumin-trends and vedolizumab pharmacokinetics for pregnancy dosing regimen optimization**

SUPPLEMENTARY MATERIAL

Zrinka Duvnjak^1,2^, Robin Michelet^1^, Casper Steenholdt^3,4^, Ella S.K. Widigson^1,2^, Cæcilie Skejø^5,6^, João A. Abrantes^7^, Wilhelm Huisinga^2,8^, Mette Julsgaard^5,6,^*, Charlotte Kloft^1,2,^*

*shared senior authorship

*^1^*Department of Clinical Pharmacy and Biochemistry, Institute of Pharmacy, Freie Universität Berlin, Berlin, Germany

*^2^*Graduate Research Training program PharMetrX, Berlin/Potsdam, Germany

*^3^*Department of Medical Gastrointestinal Diseases S, Odense University Hospital, Odense, Denmark

*^4^*Research Unit of Medical Gastroenterology, Department of Clinical Research, University of Southern Denmark, Odense, Denmark

^5^Department of Hepatology and Gastroenterology, Aarhus University Hospital, Aarhus, Denmark

^6^Department of Clinical Medicine, Health, Aarhus University, Aarhus, Denmark

^7^Roche Pharma Research and Early Development, Pharmaceutical Sciences, Roche Innovation Center Basel, Basel, Switzerland

^8^Institute of Mathematics, Universität Potsdam, Germany

**Table S1.** Comparison of covariate values used for simulations (representative pregnancy-study individual) and values used for centring of covariate effects (based on the model from Rosario et al. 2015; typical reference individual).

| **Demographics** | **Representative pregnancy-study individual** | **Typical reference individual** |
| --- | --- | --- |
| *Continuous covariates^#^* |  |  |
| Albumin [g/L] | 40 | 40 |
| Body weight [kg] | 70 | 70 |
| Faecal calprotectin [mg/kg] | 200 | 700 |
| CDAI score | 100 | 300 |
| Partial Mayo score | 1 | 6 |
| Age [years] | 30 | 40 |
| *Categorical covariates^#^* |  |  |
| Presence of antidrug antibodies | No | No |
| Diagnosis | CD | UC |
| Prior anti-TNF-α therapy use | Yes | No |

*^#^*Covariate effects are ordered by the effect size within the data type category.

Abbreviations: CDAI: Crohn's Disease Activity Index, TNF: tumor necrosis factor

**Figure S1.** Dosing regimen optimization. a) Iterative simulation process. First, only one dose was simulated, and when the concentration reached pre-pregnancy C_min_ (red horizontal line), the gestational age was recorded (blue vertical dashed line). In the next iteration, the dose was also administered at the gestational age recorded in the previous iteration. The process was repeated until the end of pregnancy, and for multiple scenarios differing in the timing of the pregnancy onset. Black arrows above the x-axis represent dosing events. b) Optimised dosing regimen (lighter colour ticks and infusion symbols) and standard (scheduled based on pre-pregnancy dosing interval) dosing regimen (darker colour ticks and infusion symbols) for two example scenarios differing in the timing of the pregnancy onset within the dosing interval (scenario 1: pregnancy onset 6 weeks after the last dose, 1^st^ pregnancy dose given at gestational age of 2 weeks; scenario 2: pregnancy onset 2 weeks after the last dose, 1^st^ pregnancy dose given at gestational age of 6 weeks). Nomogram-like plot was derived by modeling optimized dosing times as a polynomial function of the corresponding standard dosing times (assuming pre-pregnancy dosing intervals were maintained throughout pregnancy).

a


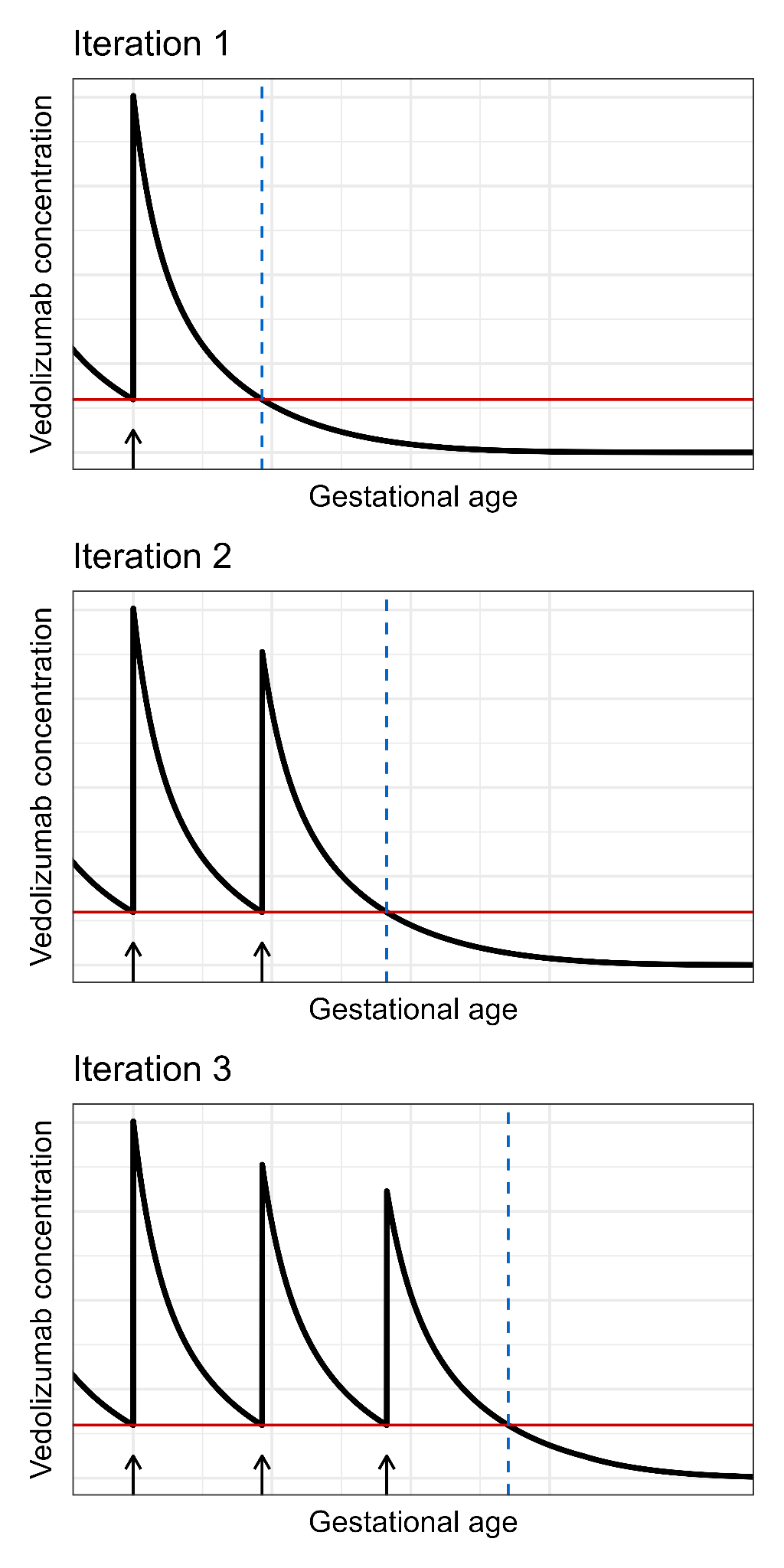


b

**
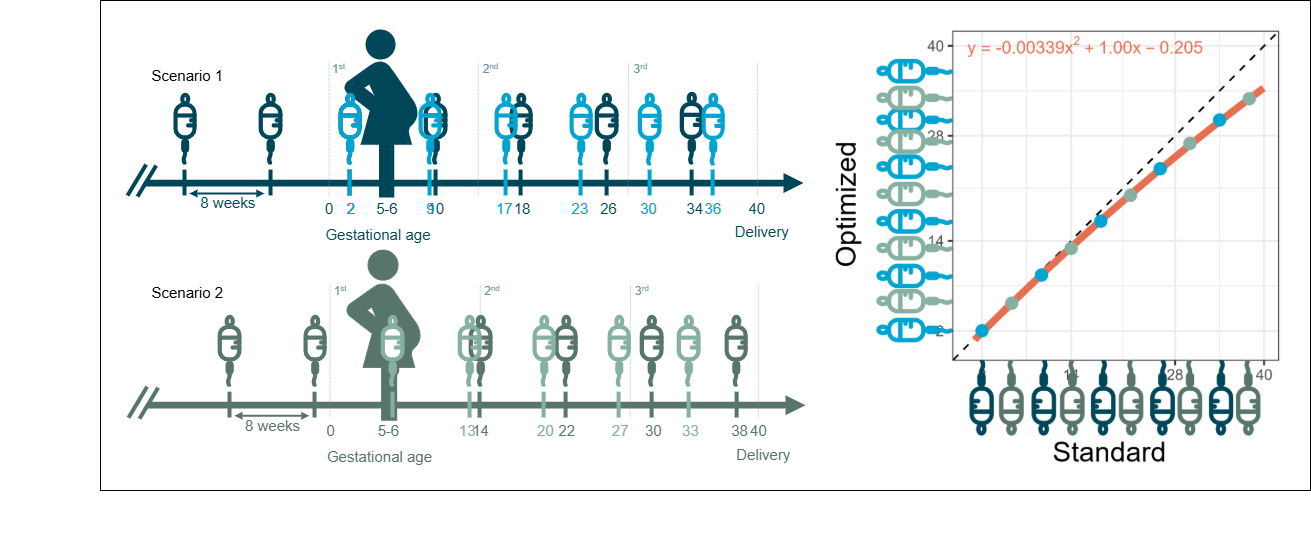
**

##

**Figure S2.** Correlation matrix for the exploratory investigation of potential covariates influencing the extent of change in albumin over time, represented by parameter B in the albumin-trends model (first line and column of the correlation matrix). Scatter plots in the lower triangle display pairwise relationships between covariates, with locally weighted smoothing lines (red) indicating potential nonlinear trends. The diagonal shows the distribution (histograms) of each covariate. The upper triangle reports Pearson correlation coefficients, with asterisks indicating the level of statistical significance (*: p < 0.05, **: p < 0.01, ***: p < 0.001).


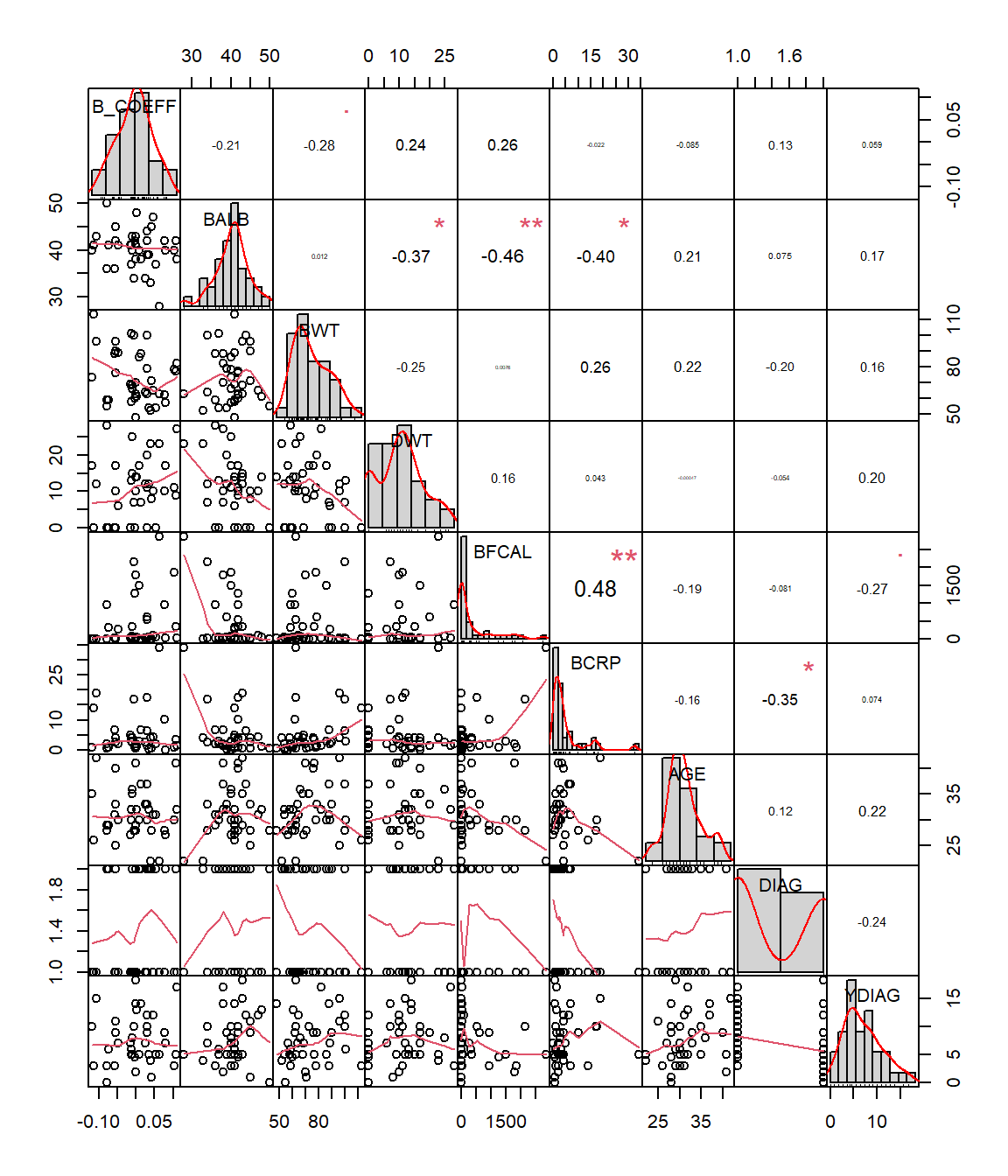


Abbreviations: B_COEFF: individual estimate of parameter B from the albumin-trends model, BALB: baseline albumin concentration, BWT: baseline body weight, DWT: body weight gained during pregnancy, BFCAL: baseline faecal calprotectin concentration, BCRP: baseline C-reactive protein concentration, AGE: maternal age at conception, DIAG: diagnosis (Crohn’s disease or Ulcerative colitis), YDIAG: years from diagnosis.

**Figure S3.** Standard goodness-of-fit (GoF) plots for the albumin-trends model. a) Conditional weighted residuals (CWRES) versus population-predicted albumin concentrations. b) CWRES versus fertilization age. c) Observed versus population-predicted albumin concentrations. d) Observed versus individual-predicted albumin concentrations. The orange line represents a locally estimated scatterplot smoothing (LOESS) fit with a shaded 95% confidence interval.


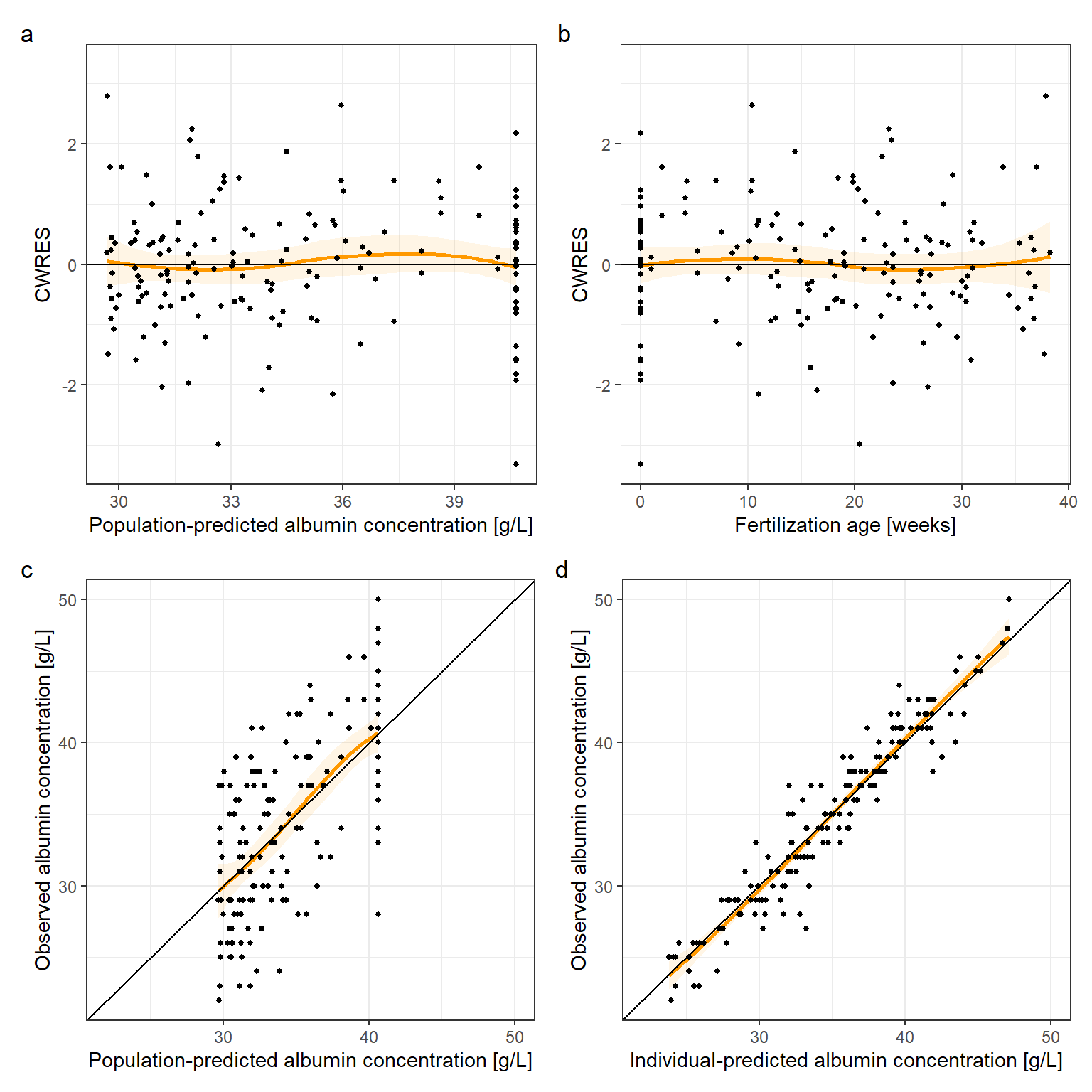


**Figure S4.** Standard goodness-of-fit (GoF) plots for the albumin trend model. a) Conditional weighted residuals (CWRES) versus population-predicted vedolizumab concentrations. b) CWRES versus time after dose. c) Observed versus population-predicted vedolizumab concentrations. d) Observed versus individual-predicted vedolizumab concentrations. The red line represents a locally estimated scatterplot smoothing (loess) fit with a shaded 95% confidence interval. One data point with CWRES=-5.66 (C_pred_=64.0 mg/L, Time after dose=10 weeks) is not included in the loess smoothing and is not shown in panels a and b.


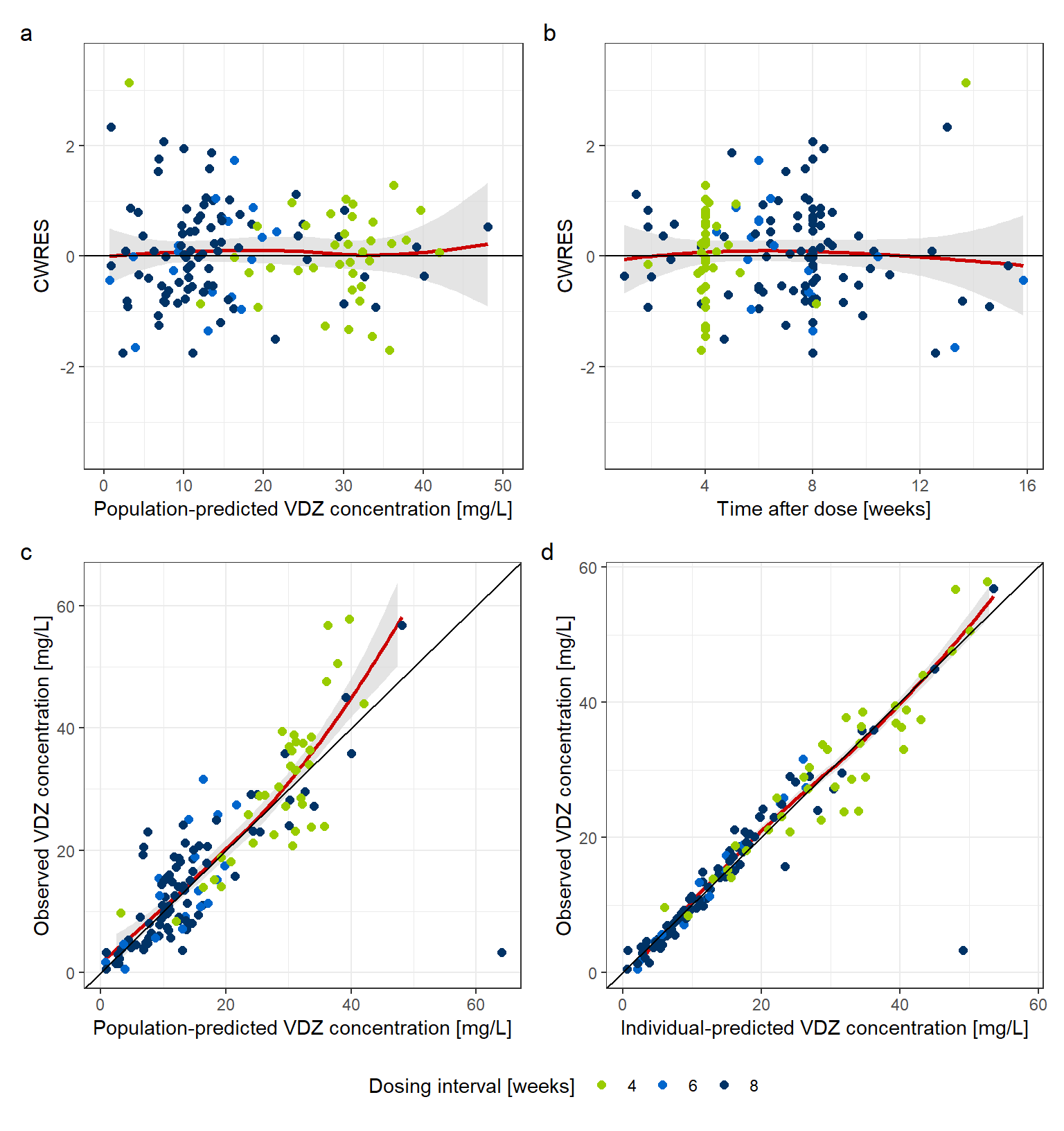


##

**Figure S5.** Covariate prediction-corrected visual-predictive check for the most relevant baseline (pre-pregnancy) covariates: a) Body weight. b) Albumin; (n=2000).

##
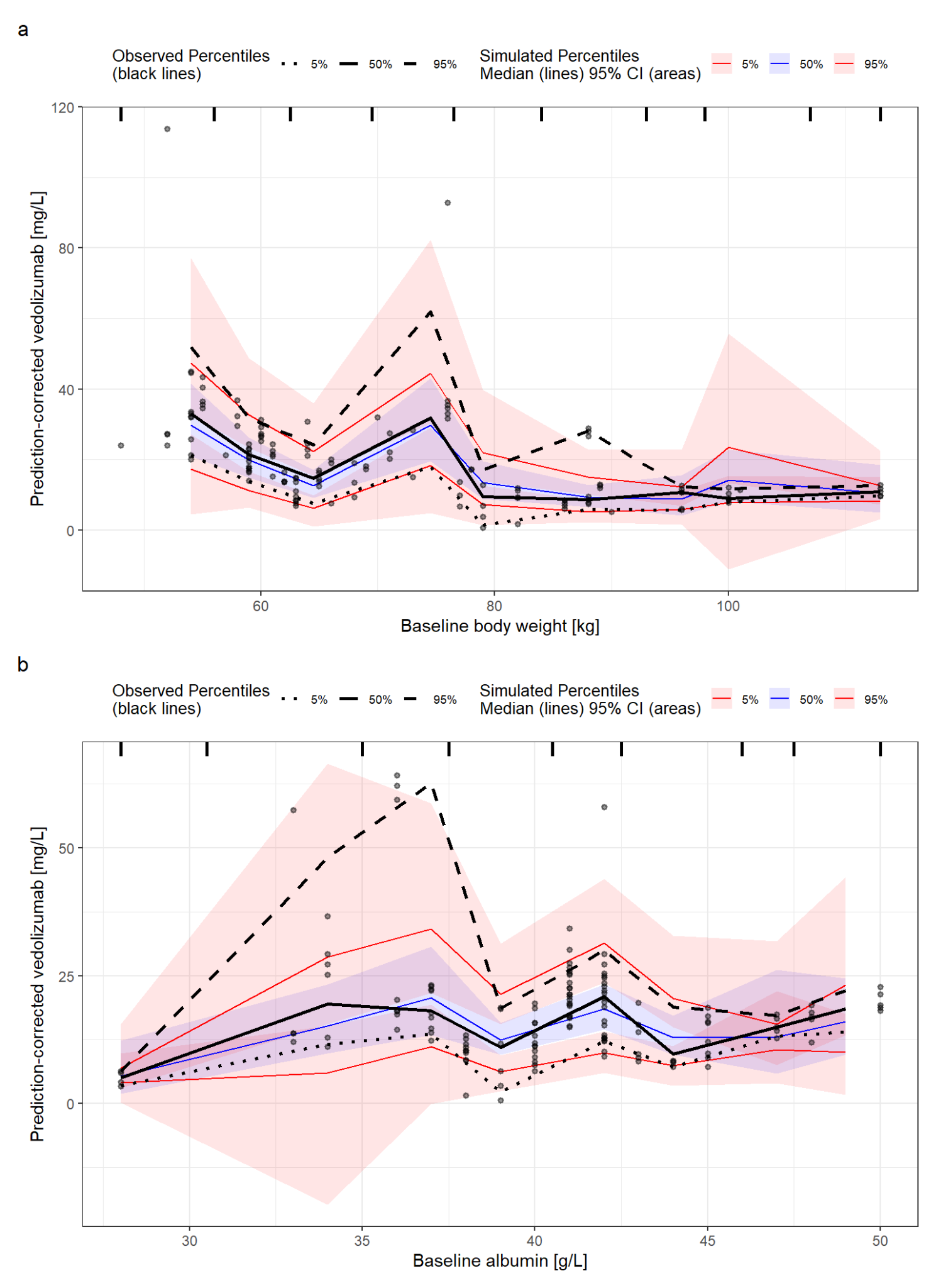


**Model code**

NONMEM code can be found in the Kloft Lab GitHub repository: https://github.com/Kloft-Lab/Duvnjak-et-al.-2025-model-code
